# GATA3 is a master regulator of the transcriptional response to low-dose ionizing radiation in human keratinocytes

**DOI:** 10.1186/1471-2164-10-417

**Published:** 2009-09-07

**Authors:** Florian Bonin, Manuella Molina, Claude Malet, Chantal Ginestet, Odile Berthier-Vergnes, Michèle T Martin, Jérôme Lamartine

**Affiliations:** 1 Université de Lyon, Lyon, F-69003, France; Université Lyon 1, Lyon, F-69003, France; CNRS, UMR5534, Centre de Génétique Moléculaire et Cellulaire, Villeurbanne, F-69622, France; 2 Centre Léon Bérard, Service de Radiothérapie, Lyon F-69008, France; 3 Laboratoire de Génomique et Radiobiologie de la Kératinopoïèse, CEA, IRCM, Evry F-91000, France

## Abstract

**Background:**

The general population is constantly exposed to low levels of radiation through natural, occupational or medical irradiation. Even if the biological effects of low-level radiation have been intensely debated and investigated, the molecular mechanisms underlying the cellular response to low doses remain largely unknown.

**Results:**

The present study investigated the role of GATA3 protein in the control of the cellular and molecular response of human keratinocytes exposed to a 1 cGy dose of X-rays. Chromatin immunoprecipitation showed GATA3 to be able to bind the promoter of 4 genes responding to a 1 cGy exposure. To go further into the role of GATA3 after ionizing radiation exposure, we studied the cellular and molecular consequences of radiation in GATA3 knock-down cells. Knock-down was obtained by lentiviral-mediated expression of an shRNA targeting the GATA3 transcript in differentiated keratinocytes. First, radiosensitivity was assessed: the toxicity, in terms of immediate survival (with XTT test), associated with 1 cGy radiation was found to be increased in GATA3 knock-down cells. The impact of GATA3 knock-down on the transcriptome of X-ray irradiated cells was also investigated, using oligonucleotide microarrays to assess changes between 3 h and 72 h post-irradiation in normal vs GATA3 knock-down backgrounds; transcriptome response was found to be completely altered in GATA3 knock-down cells, with a strong induction/repression peak 48 h after irradiation. Functional annotation revealed enrichment in genes known to be involved in chaperone activity, TGFβ signalling and stress response.

**Conclusion:**

Taken together, these data indicate that GATA3 is an important regulator of the cellular and molecular response of epidermal cells to very low doses of radiation.

## Background

The general population is constantly exposed to low levels of radiation through natural background radiation or occupational and medical activity. For example, diagnostic X-ray procedures are the main man-made source of radiation exposure, accounting for 14% of total exposure worldwide [1]. There is considerable public and scientific interest in characterizing the biological effects of ionizing radiation (IR) in the dose range occurring in the more routine X-ray procedures, with a specific focus on elucidating the underlying molecular and biochemical mechanisms.

Organs are not equally sensitive to ionizing radiation, and skin, which is the most exposed organ, is among the most sensitive. Skin is composed of three primary layers: epidermis, dermis and hypodermis. The interfollicular epidermis is a multilayered epithelium that covers the human skin. Its primary function is to serve as a barrier against the organism's environment. Keratinocytes, the main cells composing this epithelium, play a key role in the barrier function of the skin. The effects of moderate and high doses of ionizing radiation on human keratinocytes have been extensively investigated, using large scale genomic approaches [2-4]; the effects of low-dose radiation on normal keratinocytes, on the other hand, remain largely unknown. We previously [5] detected a significant transcriptional response in human keratinocytes exposed to a low dose of 1 cGy, but the molecular mechanisms regulating this response remain to be clarified. Transcription regulation is a key level of control of the cellular response to genotoxic stress. To date, little is known about the transcription factors involved in this response, especially to very low doses of ionizing radiation. To further study this question, we used a bioinformatic strategy to identify candidate transcription factors involved in the regulation of low IR dose responding genes [5]. Validating these putative regulators required a dedicated functional genomics approach, which was the goal of the present study.

The study shows for the first time that the transcription factor GATA3 binds to the promoter regions of genes responding to low IR doses, and that silencing this protein in irradiated human keratinocytes leads to generalised transcriptional deregulation after 1 cGy X-irradiation. Taken together, these data indicate that GATA3 plays a key role in the transcriptional response of epidermal cells to very low doses of radiation.

## Results

### GATA proteins bind to the promoters of 4 genes responding to low-dose radiation

**Figure 1:**
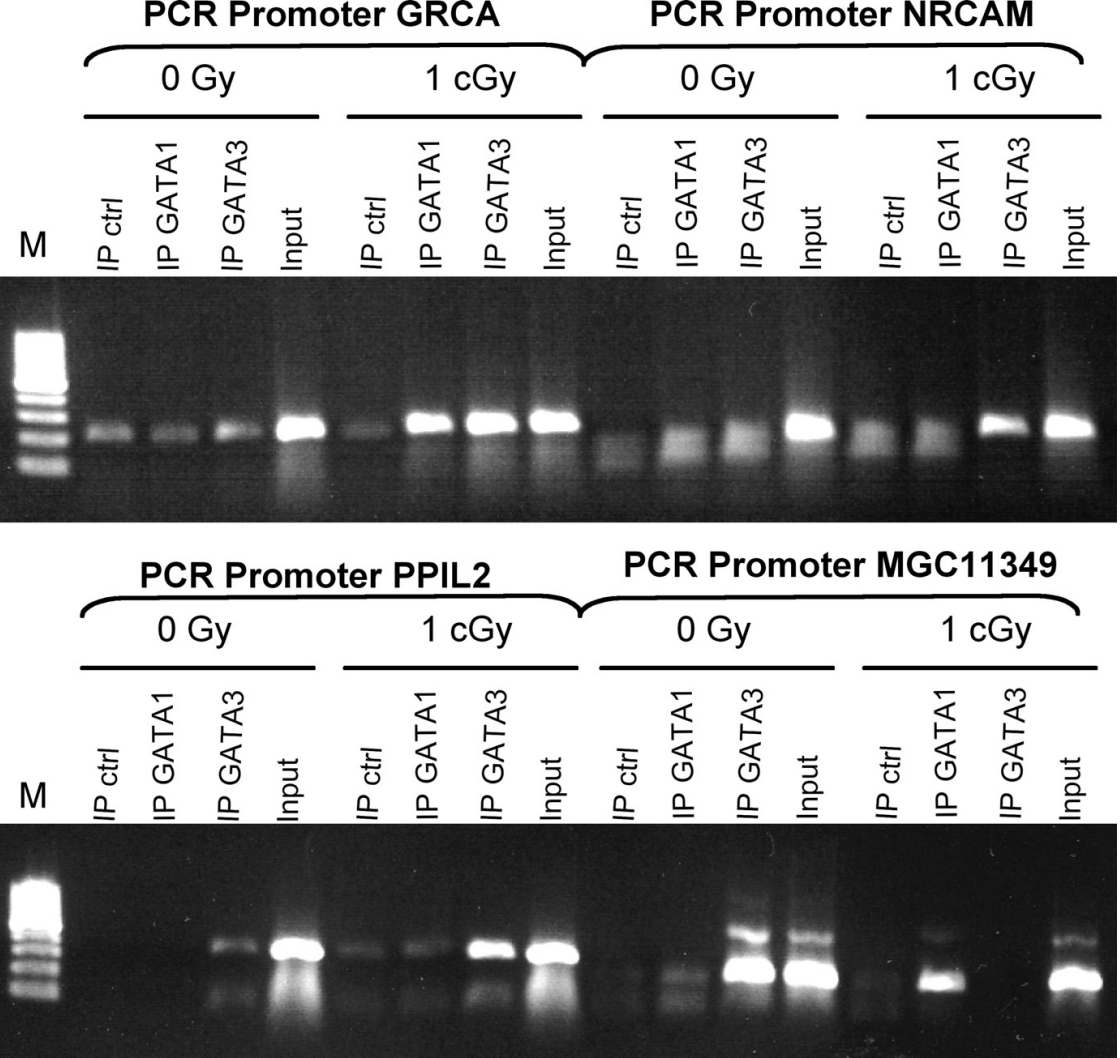
**GATA proteins bind to promoter of IR-responding genes after 1 cGy irradiation**. ChIP of primary keratinocytes with anti-GATA1 (IP GATA1) anti-GATA3 (IP GATA3) or mouse Ig (IP ctrl) after mock (0 Gy) or 1 cGy irradiation. The promoter regions of GRCA, NRCAM, PPIL2 and MGC11349 were PCR-amplified on immunoprecipitated chromatin or total input DNA and resolved on 1.5% agarose gel. M: molecular weight ladder 100 bp.

We have previously shown that low-dose IR induces a specific gene response in normal human differentiated keratinocytes [5]. A significant number of low-dose specific genes were identified, most modulated at 48 h. We focused on a cluster of 17 genes sharing a common temporal profile specific to the low dose. Bioinformatic analysis of these genes' promoter sequences revealed enrichment in GATA consensus sequences corresponding to GATA1 and GATA3 binding sites [5]. To validate the potential involvement of these transcription factors in the co-regulation of the 17 genes, we immunoprecipitated the chromatin of 1 cGy and mock irradiated keratinocytes with anti-GATA1 and anti-GATA3 antibodies. Promoter sequence enrichment was analysed in 10 of the 17 genes. PCR amplification revealed GATA3 ChIP enrichment in 3 genes of the cluster after 1 cGy irradiation (GRCA, NRCAM, PPIL2) (see Figure 1). Some of them (GRCA, MGC11349) were also found to be enriched in GATA1 ChIP (Figure 1). For MGC11349, an opposite change was observed, with loss of GATA3 binding after irradiation. Taken together, these results indicate that irradiation induces changes in GATA3 binding on the promoter sequences of these 1 cGy responding genes. GATA3 has been shown to be expressed in differentiated keratinocytes and to play a key role in skin morphogenesis [14], whereas GATA1's function appears to be restricted to the hematopoietic system [15]. Based on these data, we decided to further investigate the role of GATA3 in controlling response to low-dose radiation in human keratinocytes.

### Stable GATA3 knock-down in human keratinocytes

**Figure 2:**
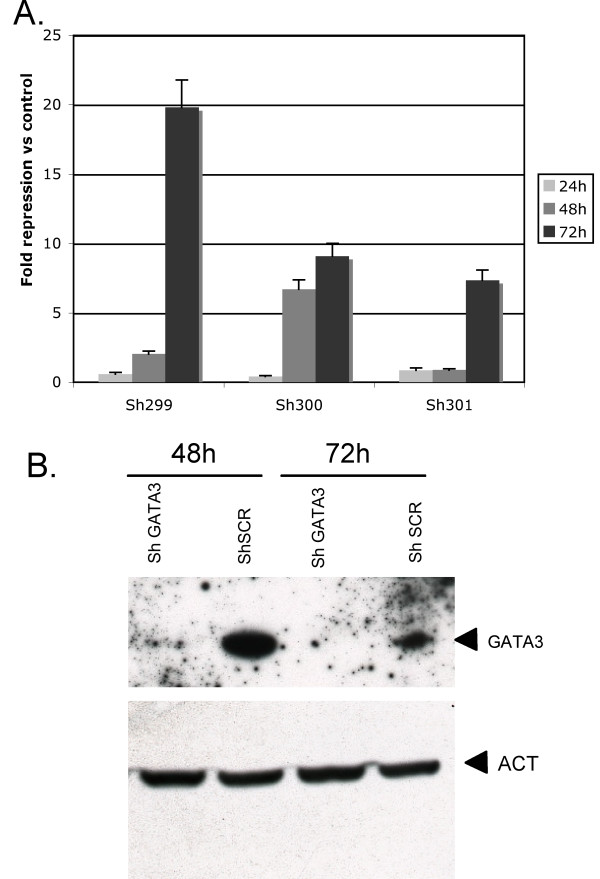
**Silencing GATA3 expression in human keratinocytes transduced with the various GATA3 shRNA vectors**. (A) Quantitative RT-PCR analysis of GATA3 transcripts at various time points after infection of human keratinocytes with the various shRNA vectors. The repression fold of the GATA3 transcript versus cells infected with the shSCR vector is indicated. (B) Analysis of GATA3 and beta-actin (ACT) protein levels by immunoblotting in human keratinocytes infected either with the sh299 (shGATA3) or the shSCR vector at 48 or 72 hours after infection.

We studied whether lentiviral vector-expressed shRNA could silence GATA3 expression in differentiated human keratinocytes. Three individual shRNAs targeting different portions of GATA3 mRNA were tested in cultured keratinocytes. The knock-down efficiency of each individual shRNA was measured by RT-PCR at various times after infection (Figure 2A). All three shRNAs caused significant repression of GATA3 mRNA, but sh299 was the most efficient, with a 20-fold decrease 72 hours after infection. At protein level, in cells expressing sh299, a marked decrease in GATA3 protein levels was observed at 48 h and 72 h (Figure 2B). This shRNA clone was therefore selected for a large-scale production of lentiviral particles that were used to establish stable GATA3 knock-down after puromycin selection of infected keratinocytes. A lentiviral vector expressing an shRNA without any target in the human genome (shSCR) served as control.

### Radiosensitivity of keratinocytes expressing shGATA3

**Figure 3:**
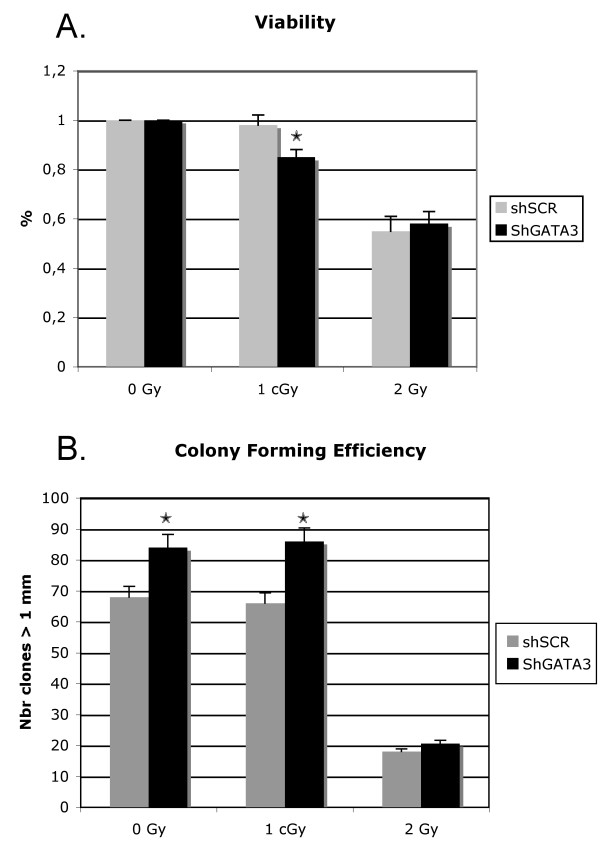
**Radiosensitivity of shGATA3 keratinocytes**. (A) Short-term radiosensitivity: Transduced keratinocytes were exposed to 1 cGy or 2 Gy radiation, and viability was measured after 72 h by the XTT method. Results are expressed as the mean percentage +/- SD of viable cells of 3 independent experiments, assuming 100% viability for non-irradiated cells. *p < 0.05. (B) Long-term radiosensitivity: Transduced cells were plated at low density (20 cells/cm^2^). The next day, they were irradiated at 1 cGy or 2 Gy and then cultured for 2 weeks. The number of colonies obtained is expressed as the mean percentage +/- SD of 3 independent experiments. *p < 0.01.

The radiation sensitivity of keratinocytes expressing shGATA3 and shSCR was assessed by proliferation-based assay (XTT assay). Cell viability was monitored 72 h after irradiation, since it is known that cell death may take at least two days to occur in irradiated keratinocytes [10]. ShGATA3 cells were found to be more sensitive than shSCR cells at 1 cGy, whereas no significant difference was observed after a dose of 2 Gy (Figure 3A). This result highlights a specific role of GATA3 transcription factor in the early response to the lower dose. To evaluate long-term radiosensitivity, colony formation assay was used. Cells were seeded at low density, then irradiated the next day at either 2 Gy or 1 cGy and cultured for 2 weeks. The number of colonies was then determined by manual counting (Figure 3B). More colonies were found for shGATA3 cells, whatever the radiation dose. This could reflect the overall positive effect of GATA3 knock-down on cell proliferation, previously described in epithelial cells [10,16]. This effect is similar in both 0 Gy and 1 cGy irradiated cells, indicating that long-term radiosensitivity is unchanged in shGATA3 cells after 1 cGy irradiation.

### Transcriptome analysis of shGATA3 and shSCR cells

**Figure 4:**
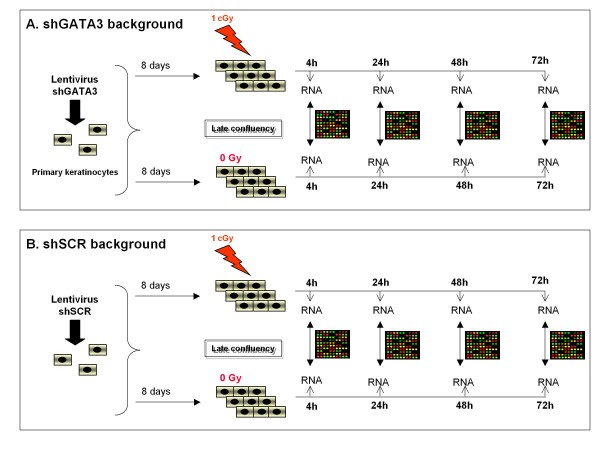
**Experimental strategy of the transcriptome analysis**.

To compare the response of shGATA3 and shSCR cells to low-dose radiation, we set up a large-scale transcriptome analysis using oligonucleotide microarrays. The experimental strategy is depicted in Figure 4. In brief, primary human keratinocytes were cultured in a semi-defined medium and then infected with either sh299 (Figure 4A) or shSCR lentiviral particles (Figure 4B). Cells were then cultured for 5 days up to confluence and then further incubated for 3 additional days before being subjected to a 1 cGy dose of X-rays. Total RNA was extracted 4, 24, 48 or 72 hours after irradiation. After RNA amplification and labelling, gene profiling was performed using oligonucleotide microarrays (26,068 probes) to compare 1 cGy irradiated to sham-irradiated cells at individual times. Two similar analyses were performed: one in cells where GATA3 was knocked down after expression of the sh299 sequence (shGATA3 background; figure 4A) and one in control cells expressing the shSCR sequence (shSCR background; figure 4B). The silencing of GATA3 in the shGATA3 background was checked by qRT-PCR (see Additional File 1).

For data analysis, Lowess normalization was applied; genes were selected on a p-value of p < 0.01 and the induction/repression cut-off values were 1.2 and 0.8, respectively.

**Figure 5:**
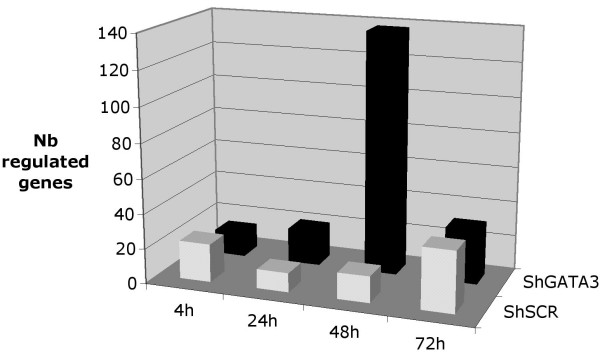
**Gene modulation at individual time points in shGATA3 and shSCR transduced cells**.

Firstly, the number of genes induced or repressed by the 1 cGy radiation was compared between the shGATA3 and shSCR backgrounds. In the shSCR background, the number of genes significantly induced or repressed after 1 cGy X-irradiation was relatively small (< 30) at all time points, and most transcripts appeared to be modulated less than 2-fold (Figure 5). In the shGATA3 background, a burst of radiation-responding genes was observed, with 266 genes (174 induced, 92 repressed) transcriptionally regulated 48 h after irradiation. Moreover, the magnitude of the change was completely modified, with abundance more than doubled for 50 of the 266 genes. At the other shGATA3 analysis time points, the number of regulated genes was comparable to that in the shSCR analysis. (See Additional file 2 to 9 for the lists of IR-responsive genes at each time point for both cell cultures).

**Figure 6:**
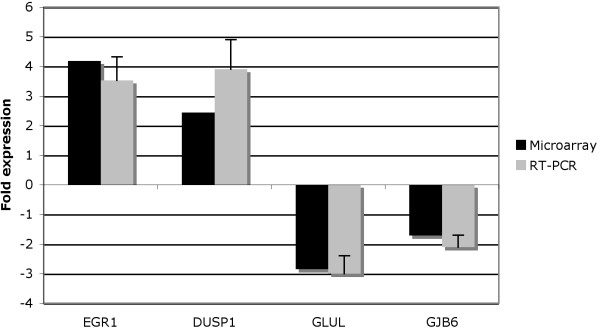
**Comparison of microarray and real-time PCR experiments**. For 4 genes modulated in shGATA3 cells, microarray ratios were compared to real-time PCR ratios. The given ratios are the expression levels of irradiated compared to non-irradiated cells. RT-PCR ratios are the means of 3 independent amplifications. Errors bars correspond to standard deviations.

To validate the microarray results, two induced genes (EGR1, DUSP1) and two repressed genes (GLUL, GJB6) at 48 h in shGATA3 cells were selected and further studied by real-time quantitative PCR. The transcriptional response was confirmed in all 4 genes (Figure 6). As described previously [2], the induction ratios on real-time PCR were often stronger than those obtained by the microarray experiments.

### Functional annotation and promoter analysis of IR-responding genes

Genes were classified into functional groups on the basis of biological process categories from the Gene Ontology Consortium [17]. Total gene lists responding to 1 cGy IR were compared to all analysable genes in the array, using the categorical over-representation function of DAVID. Owing to the small number of IR-responding genes in most of the conditions, only genes up-regulated at 48 h in shGATA3 cells gave highly significant enriched functional categories (see Table 1). The key up-regulated biological processes in shGATA3 cells were found to be chaperone activity, TGFbeta signalling, stress response, RNA metabolic process and phosphatase activity. At the other time points, several functional groups, including protein-protein interaction and stress response, were also enriched, with scores less than 0.01 (data not shown).

**Table 1:** Functional annotation of shGATA3-regulated genes at 48 h

	**Biological Processes**	**EASE Score**	**Nb of Genes annotated**	**Genes Lists**
**48 h shGATA3 induced**	Chaperone	2.3 E-5	9 out 174	CMT2L, CANX, HSPA9B, HYOUN1, PTGES3, TIMM9, PFDN2, TRAP1, TXNDC4
	TGFb signaling	2.1 E-5	7 out 174	TGIF1, TSC22D1, TGFBR1, INHBE, SMAD4, ID1, E2F5
	Phosphatase activity	5.3 E-4	3 out 174	UBLCP1, DUSP1, PTPN12
	Response to stress	5.3 E-4	14 out 174	DUSP1, EGR1, CMT2L, HSPA9B, HYOUN1, DDIT3, PRNP, SFPQ, NFATC3, SYVN1, TRAP1, ACVRL1, NCR3, HBEGF
	RNA metabolic process	8.7 E-3	18 out 174	DDIT3, PPP2R1B, ELL2, EGR1, NFATC3, SFPQ, E2F5, ID1, NFT3, TSC22D1, ZNF146, SF3B14, WDSOF1, ZNF238, ZFP3, CGGBP1, SMAD4, HMGN2
**48 h shGATA3 repressed**	Cytoplasm	2.2 E-3	39 out 92	*

The overrepresented Gene Ontology categories determined by EASE are indicated. * Due to the high number of annotated genes in the 48 h shGATA3 repressed line, the 39 genes are not listed.

We then focused on the genes transcriptionally deregulated 48 h after irradiation in shGATA3 cells. To determine whether these genes might be direct targets of GATA3, a bioinformatic analysis of their promoter sequences was performed. The goal was to assess their putative enrichment in GATA3 binding sequences. The promoter sequences (-750, +250 with respect to the Transcription Start Site) of the 30 most induced and most repressed genes in shGATA3 cells at 48 h were studied using the MatInspector promoter analysis tool [13]. As a control, a random sample of 50 genes was also analysed. Genes repressed at 48 h in shGATA3 cells exhibited two-fold enrichment in GATA3 consensus sequences compared to induced genes and the random control sample (Table 2). These results indicate that the list of genes repressed in shGATA3 cells might be enriched in direct GATA3 targets, whereas induced genes are probably indirectly regulated by GATA3. The fact that two of the targets validated by ChIP (Figure 1), GRCA and PPIL2, are among these repressed genes reinforces this hypothesis.

**Table 2:** Promoter analysis of the IR-responding genes in shGATA3 cells at 48 h.

	**Induced**	**Repressed**	**Random sample**
**GATA3 sites/promoter**	0.31 +/- 0.02	0.72 +/- 0.08	0.28 +/- 0.03

Mean number of putative GATA3 binding sites in the promoter sequences (-750, +250) of the 30 most induced and 30 most repressed genes in shGATA3 cells. The mean number of putative GATA3 binding sites is indicated. As a control, a random sample of 50 non-responding genes was also analyzed.

## Discussion

GATA3 is a zinc finger transcription factor essential for the proper development of various tissues and organs, especially in the hematopoietic system. GATA3 has been recently shown to be involved in the development of hair follicles and in the determination of skin cell lineage in mice [14,18]. In human skin, GATA3 is expressed in suprabasal layers of the epidermis [19] and is also regulated during the differentiation of cultured keratinocytes: GATA3 accumulates during keratinocyte differentiation induced by addition of calcium [19]. GATA3 is therefore strongly expressed in differentiated cultured keratinocytes, which were the cell models used in our previous transcription study [5]. GATA3 plays a key role in the transition between proliferation and differentiation in human epidermal cells through the p63/IKKα/SMAD pathway [19,20], but its function in the control of the cellular response to genotoxic stress has never been explored.

To monitor the function of GATA3 in the low-dose IR response, we set up a cell model of primary keratinocytes with stable knock-down of GATA3. We decided to study cultured keratinocytes in a differentiated state mimicking the suprabasal layer of human epidermis, since these cells are the first to be exposed in therapeutic or accidental irradiation. GATA3 was knocked down by a lentiviral-mediated shRNA expression. As previously reported by others [21], lentiviral vectors appear to be the most suitable for long-term transgene expression, especially in quiescent cells such as stem cells or confluent keratinocytes, whereas chemical methods or electroporation are completely inefficient. We were able to infect more than 70% of our cultured cells and obtained a marked decrease in GATA3 protein level, which became almost undetectable on western-blot, in infected cells. What are the biological consequences of this knock-down? In a normal context, keratinocytes infected by the shGATA3 vector exhibit a higher rate of proliferation (J. Lamartine. In preparation). This effect was clearly visible in our colony-forming assay, where shGATA3 cells were found to be more clonogenic than shSCR cells, whatever the radiation dose. It can therefore be postulated that this long-term effect on proliferation conceals shGATA3 cell hyper-sensitivity to certain doses of radiation. Indeed, the XTT assay, performed 72 h after exposure, revealed specific shGATA3 cell radiosensitivity to the 1 cGy dose, with a 16% decrease in cell viability compared to shSCR cells. After the 2 Gy dose, both cell lines exhibited the same viability. These results indicate that silencing GATA3 modifies the cellular response of irradiated cells, specifically to the low 1 cGy dose, during the first hours following irradiation. Specific radiosensitivity to low IR doses has been previously described for human keratinocytes [22,23]. Low-dose hyper-radiosensitivity (HRS) is an effect in which cells die from excessive sensitivity to low doses (< 0.5 Gy) while becoming resistant to higher doses. The suggested mechanism for HRS is related to the absence at low doses of the inducible DNA repair mechanism observed at higher doses [24]. Our data indicate that GATA3 knock-down caused increased cell death after 1 cGy IR compared to shSCR cells, highlighting the important role of this protein in the 1 cGy response during the first 72 hours following irradiation. To more closely delineate the relative role of low-dose HRS in this increased cell death, additional studies using a larger range of dose between 1 cGy and 0.5 Gy will be useful.

To further test the role of GATA3, we compared the transcriptional response of shGATA3 and shSCR cells after 1 cGy IR exposure: a modified gene response profile was observed, with a burst of IR-responding genes in shGATA3 cells at 48 h. This time-point of 48 h seems to be a key moment in the transcriptional response. Our previous microarray studies found that the response to 1 cGy, contrary to the classical bimodal response after higher doses, was characterized by almost complete absence of transcriptional changes at early time points, followed by a large modulation at 48 h post-irradiation [5]. It can be postulated than GATA3 is involved in the control of this 48 h gene response.

Many of the 266 genes responding at 48 h in shGATA3 cells were involved in fundamental mechanisms known to participate in the cellular response to genotoxic stress. Such is the case of EGR1, the gene showing the strongest induction ratio (> 4-fold). EGR1 is a transcription factor strongly activated by a broad spectrum of radiation, and promoting apoptosis and growth arrest through its targets, especially some members of the p53 families and via activation of the EGFR/ERK1/2 pathway [25]. The DUSP1 gene, encoding a dual-specific threonine and tyrosine phosphatase, is also strongly induced in shGATA3 cells. DUSP1 is controlled by p53 during the cellular response to genotoxic stress and is a potent inhibitor of MAPK activity through dephosphorylation of MAPK [26]. The functional annotation of IR-responding genes in shGATA3 cells (Table 1) revealed several biological processes known to participate in the cellular response to stress: this is the case with TGFβ signalling, which concerns 7 of the 174 genes induced at 48 h in shGATA3 cells (TGIF, TSC22D1, TGFBR1, INHBE, SMAD4, ID1, E2F5). TGFβ1 is involved in the initiation of keratinocyte differentiation, by blocking their proliferation. This cytokine is also involved in wound healing, by inducing cell migration and keratinocyte matrix secretion [27]. Moreover, TGFβ1 is induced in skin within hours following acute irradiation [28] and plays a complex role in regulating the canonical cellular DNA damage response through ATM activation [29]. A possible link between TGFβ1 expression and regulation by GATA proteins has been proposed in hematopoietic cell systems, but the involvement of GATA3 in the regulation of the TGFβ pathways has never been described in keratinocytes. Our functional annotation of IR-regulated genes in shGATA3 cells also revealed enrichment in genes involved in chaperone activity and protein folding. Molecular chaperones prevent protein aggregation and keep proteins in a state suitable for either refolding or degradation after a proteolytic stress such as ionizing radiation [30]. The present study showed that GATA3 knock-down led to excessive sensitivity to low IR doses. It is possible that, in these cells, the fraction of proteins in an unfolded state is increased after irradiation, inducing the chaperone response.

The central question raised by our study is the link between GATA3 and the genes that are deregulated in shGATA3 cells. To answer this question, we looked for GATA consensus sites in the promoter region of genes that are induced or repressed after irradiation in shGATA3 cells, as compared with a random sample of non-responding genes. We did not observe any enrichment in the fraction of genes that are up-regulated: these genes are probably not direct targets of GATA3. Their up-regulation appears to be secondary to knock-down, or a specific response of cells sensitized to low-dose radiation. On the other hand, the frequency of GATA3 binding sites increased in the fraction of down-regulated genes (Table 2). Some of these genes, such as PPIL2 and GRCA, which we have shown to be bound in vivo by GATA3 (figure 1), might be direct targets of GATA3. Nevertheless, further exploration of the GATA3 targets in the low-dose IR response will be necessary to clarify this point. The recent development of methods allowing genome-wide identification of transcription factor targets, such as ChIP chip [31] or ChIP seq [32], will offer the possibility of achieving this goal.

## Conclusion

Over the last few years, numerous large-scale transcription studies have been set up in various cell types to identify radiation-responsive genes that could serve as biomarkers [33]. Even though hundreds of differentially expressed transcripts have thus been listed, the molecular mechanisms controlling the transcriptional regulation of these genes remain unknown. We demonstrate here for the first time the involvement of a transcription factor in the response of human cells to very low doses of X-radiation, and we show that various pathways and cellular functions are directly or indirectly dependent on this regulator. These findings open the door to better understanding of the biological effects of extremely low-dose ionizing radiation on human cells.

## Methods

### Cell culture

Human keratinocytes were isolated from neonatal foreskin after routine circumcisions. A written informed consent was obtained from the infant's parents according to the French bioethical law of 2004 (loi 94-654, 29 July 1994) and the guidelines of the Helsinki Declaration. Keratinocytes were isolated and cultured as previously described [5]. Briefly, after isolation by overnight trypsinization, cells were cultured in the semi-defined KGM2 medium (Lonza) on flasks coated with collagen type I (Falcon Biocoat) at 37°C and 5% CO2. Our model of study consisted of human keratinocytes grown to confluence to induce a differentiation program mimicking the suprabasal layer of the epidermis. To obtain such a differentiation state, second passage cultures were seed at 20000 cells/cm2, reached confluence between day 5 and 8 depending on the keratinocyte preparation, and then further incubated for 3 days post-confluence.

### Production of lentiviral particles and cell transduction

The sh-RNA expressing lentiviral vectors (pLKO.1-puro-shRNA) were purchased from Sigma (Mission shRNA library). Three plasmid clones expressing a shRNA targeting the GATA3 mRNA (pLKO.1-puro-sh299, sh300, sh301), as well as a non-target shRNA control vector (pLKO.1-puro-shSCR) were obtained and used to transfect HEK293T cells along with third-generation lentivirus packaging and pseudo-typing plasmids as described by Wu et al [6]. The viral supernatant was collected 48 h after transfection and concentrated by using a Centricon Plus-70 filter unit (Millipore). The virus vector particles were then used to transfect semi-confluent cultured keratinocytes at a multiplicity of infection (MOI) of 5 particles/cell in KGM2 medium in 6 well plates. Cells with stable integration of the shRNA construct were isolated through puromycin selection, and a pooled population of clones was used for all studies. The sequence of the shRNA targeting GATA3 (sh299) used in our studies is the following: 5'CCGGGCCAAGAAGTTTAAGGAATATCTCGAGATATTCCTTAAACTTCTTGGCTTTTT 3'

### Irradiation

The irradiation has been delivered by a 6 MV photon beam produced by a linear accelerator (Elekta Precise; Elekta, Crawley, UK). The cells dishes were placed on a water-equivalent phantom of 5 cm thickness to generate an enough back scattered radiation. The gantry of the linear accelerator was positioned at 0 degree. The cells dishes were positioned on this phantom with 2 cm of a water-equivalent phantom above. We used these conditions to measure and validate the dose with a wall ionisation chamber (Markus, PTW, Freiburg, Germany), and the distance (108 cm) between the source and the chamber was adjusted just to obtain 1MU = 1cGy.

### Chromatin Immunoprecipitation

For ChIP assay, human keratinocytes cultured to post-confluence in KGM2 were irradiated (mock or 1cGy irradiation) and then incubated again for 48 hours. ChIP assays were performed on 10^7 ^cells as previously described [7]. Briefly, after fixation in 1% formaldehyde, cells were lysed for 5 minutes in 50 mM tris, pH 8.0, 2 mM EDTA, 0.1% NP-40 and 10% glycerol supplemented with anti-protease (Roche). Nuclei were re-suspended in 50 mM Tris, pH 8.0, 1% SDS, 5 mM EDTA. Chromatin was sheared by sonication. After pre-clearing with protein A beads (Santa Cruz), lysates were incubated overnight at 4°C with anti-GATA3 (sc-269, Santa Cruz Biotehcnology, Inc), anti-GATA1 (sc-269, Santa Cruz Biotechnology, Inc), or anti-mouse immunoglobulins (Santa Cruz Biotechnology, Inc). Immune complexes were collected with protein A, washed three times with high salt buffer (HEPES/KOH 50 mM, NaCl 140 mM, EDTA 1 mM, triton X-100 1%, sodium deoxycholate 0.1%), two times with low salt buffer (Tris-Hcl 10 mM, LiCl 250 mM, EDTA 1 mM, NP40 0.5%, sodium deoxycholate 0.1%) and then two times with Tris/EDTA (TE). Immune complexes were extracted in 1× TE buffer, and protein crosslink was reverted by heating at 65°C for 5 hours. DNA was then extracted by phenol-chloroform, ethanol precipitated and a 1/20 fraction of the immunoprecipitated DNA was used in each PCR reaction. PCR reactions were performed for 35 cycles at 95°C for 30 seconds, annealing at 60°C for 30 seconds, and extension at 72°C for 45 seconds. The following primers were designed to amplify the promoter sequences of putative GATA3 targets:

PPIL2f: 5'-ATACTGGGCAGGCGATCCTT-3'

PPIL2r:5'CGAGTTCCGTGACCACTTCC-3'

GRCAf: 5'-CCACTTCCCCACCTCACTTA-3'

GRCAr: 5'GATCCCCTAGAGGCCAGAAC-3'

NRCAMf:5'-GCATCCGCCTTATGCTAAA-3'

NRCAMr: 5'-TGTGGGAGGTCTGTGGTCT-3'

MGCf: 5'-GAGTGCCCTGTTTTCCCATA-3'

$MGCr: 5'-AATCTTGGGTCCAAATGCTG-3'

### Real-time PCR

For expression analysis of the GATA3 transcript, total RNA was extracted 24 h, 48 h or 72 h after lentiviral infection using the classical Tri-Reagent protocol (Sigma). For each condition, 500 ng of total RNA were reverse-transcribed using the RevertAid H minus M-MuLV enzyme as per the manufacturer's recommendations (Fermentas). Real-time PCR were performed with a LightCycler^© ^2.0 real-Time PCR system (Roche) using the following primers for the GATA3 transcript: GATA3forward 5'-ATACACCACCTACCCGCCTAC-3' GATA3reverse: 5'-ACTCCCTGCCTTCTGTGCT-3'. For validation of IR-responding genes, the following oligos were used: GLULf: 5'-AAACTAAGCAAGCGGCACCA-3' GLULr: CACCAGCAGAAAAGTCGTTGA-3'; DUSP1f 5'-TGGAGGAAGGGTGTTTGTCC-3' DUSP1r 5'-TGAAGTTGGGAGAGATGATGC-3'; EGR1f 5'-GTTTGCCAGGAGCGATGAAC-3' EGR1r 5'-GGGGACGGGTAGGAAGAGAG-3', GJB6f 5'-TTCATCGGGGGTGTCAACAAA-3' GJB6r 5'-GCAGACGAAGTCCTCTTGCTC-3'. Data were normalized to the 18S rRNA internal standard. Fold differences were calculated using the mathematical model described by Pfaffl [8].

### Protein extraction and western-blotting

Protein from shRNA transduced keratinocytes were extracted using RIPA buffer (1% NP40, 0.5% Sodium deoxycholate, 0.1% SDS) containing protease inhibitor cocktail (Complete Mini; Roche Diagnostics). Protein concentration were assayed using the DC protein assay kit (BioRad). Heighty micrograms of total extracted protein was fractionated on 10% SDS-polyacrylamide gel, electrophoretycally transferred to a PVDF membrane, and blocked one hour at room temperature in TBS-1% Tween20-10% powdered milk. GATA3 immunoreactivity was detected using a mouse monoclonal anti-GATA3 antibody (sc-268, Santa Cruz Biotechnology, Inc) and a peroxidase-conjugated goat anti-mouse antibody (Calbiochem) and visualized by enhanced chemiluminescence (ECL; GE Healthcare Life Sciences) according to the manufacturer's instructions.

### Short-term radiosensitivity: XTT assay

Cell viability was determined by the modified XTT assay, a test designed to measures the activity of the succinate-tetrazolum reductase, a mitochondrial enzymatic system which is active only in viable cells [9]. Briefly, 5000 cells transduced either with the shGATA3 or shSCR lentiviral vector were plated in 96-well plates with 100 microliters of KGM2, the next day the medium was changed and the cells were exposed to 2 Gy or 1 cGy of X radiation. The irradiated cells were then incubated 72 hours and then the viability was assessed using the XTT test (Sigma) exactly as described [10].

### Long term radiosensitivity: colony-forming assay

To determine the long-term radiosensitivity, an *in vitro *colony-forming assay was performed. Equivalent numbers of keratinocytes previously transduced with the lentiviral vectors expressing either shGATA3 or shSCR sequences were seeded at low density (20 cells/cm^2 ^to 100 cells/cm^2^) in 100 mm culture dishes. The next day, the plates were irradiated (1 cGy or 2 Gy). Cells were then cultured in KGM (Lonza) for 2 weeks. Keratinocytes colonies were then fixed with ethanol and stained with Mayer's Hemalun for 15 minutes (Merck) before water washing, and drying. Colonies larger than 1 mm were counted in 3 plates for each experimental condition.

### DNA microarray hybridation and analysis

Total RNA from irradiated keratinocytes was extracted using the RNAeasy kit (Qiagen) at various time after irradiation (see Figure 4). Extracted RNA was further amplified using the AminoAllyl MessageAmp II aRNA amplification kit (Ambion,) following the instructions provided by the manufacturer. For each hybridization, 1 μg of amplified aRNA from irradiated and control cells was labeled by an indirect method using monofunctional NHS-ester Cy3 or Cy5 (GE Healthcare Life Sciences). The hybridization of the microarrays was performed on two slides for each time-point in a dye-swap procedure as described [2]. We used microarrays spotted with 26 068 long oligos (50 mer) representing 21 000 human genes [11]. Slides were scanned using a Genepix 4000B microarray scanner (Axon Instrument, Molecular Devices). For each hybridized spots, the Cy3 and Cy5 fluorescence values were obtained by using Genepix Pro 6.0 software (Molecular Devices) and saved as a result file. Spots or areas of the array with obvious blemishes were manually flagged and excluded from subsequent analysis. Results files were imported into Genespring GX 7.3.1 (Agilent) for further analysis. To eliminate dye-related artifacts in 2 color experiments, intensity-dependent Lowess normalization was performed. Statistical significance of expression ratios was calculated using the Student's t test. Differentially expressed genes were selected as described in the Results section. All the microarray data have been deposited into the GEO database at  and are available under the accession number GSE15716.

### Functional annotation and promoter analysis

The sets of differentially expressed genes were further analyzed for functional significance using the DAVID bioinformatics resources at . This software obtains the Gene Ontology (GO) annotations from a database and generates a statistical analysis of the functional annotations that are overexpressed in the imputed list of genes [12], with a Bonferroni correction for multiple comparisons included. GO processes with score less than 0.05 were considered to be statistically significant. For promoter analysis, promoter sequences (-750, +250) were retrieved from the promoter database at  and screened for GATA3 consensus sites using the MatInspector promoter analysis tool [13].

## Authors' contributions

FB performed most of the experiments (lentivirus transfection, qRT-PCR, radiosensibility analysis and microarray hybridization). MM carried out keratinocytes culture from skin tissues. CG and CM settled up the radiation system and performed dosimetric measures. OB participated in cell culture and revised the manuscript. MTM participated in the design of the study and gave textual advice. JL conceived the study, performed microarray normalization and data analysis, and drafted the manuscript. All the authors read and approved the final manuscript.

## Supplementary Material

Additional file 1 **Relative expression of GATA3 in shSCR and shGATA3 non-irradiated cells**. Quantitative RT-PCR analysis of GATA3 transcripts at various time points after mock-irradiation of human keratinocytes with the various shRNA vectors. Errors bars correspond to standard deviations. Click here for file

Additional file 2 **List of genes induced and repressed at 4 h in shSCR cells**. Genes were selected on a p-value of p < 0.01 and with an induction/repression cut-off values of 1.2 and 0.8, respectively. In the table, the expression ratio of irradiated versus non-irradiated cells is indicated. Click here for file

Additional file 3 **List of genes induced and repressed at 24 h in shSCR cells**. Genes were selected on a p-value of p < 0.01 and with an induction/repression cut-off values of 1.2 and 0.8, respectively. In the table, the expression ratio of irradiated versus non-irradiated cells is indicated. Click here for file

Additional file 4 **List of genes induced and repressed at 48 h in shSCR cells**. Genes were selected on a p-value of p < 0.01 and with an induction/repression cut-off values of 1.2 and 0.8, respectively. In the table, the expression ratio of irradiated versus non-irradiated cells is indicated. Click here for file

Additional file 5 **List of genes induced and repressed at 72 h in shSCR cells**. Genes were selected on a p-value of p < 0.01 and with an induction/repression cut-off values of 1.2 and 0.8, respectively. In the table, the expression ratio of irradiated versus non-irradiated cells is indicated. Click here for file

Additional file 6 **List of genes induced and repressed at 4 h in shGATA3 cells**. Genes were selected on a p-value of p < 0.01 and with an induction/repression cut-off values of 1.2 and 0.8, respectively. In the table, the expression ratio of irradiated versus non-irradiated cells is indicated. Click here for file

Additional file 7 **List of genes induced and repressed at 24 h in shGATA3 cells**. Genes were selected on a p-value of p < 0.01 and with an induction/repression cut-off values of 1.2 and 0.8, respectively. In the table, the expression ratio of irradiated versus non-irradiated cells is indicated. Click here for file

Additional file 8 **List of genes induced and repressed at 48 h in shGATA3 cells**. Genes were selected on a p-value of p < 0.01 and with an induction/repression cut-off values of 1.5 and 0.65, respectively. In the table, the expression ratio of irradiated versus non-irradiated cells is indicated. Click here for file

Additional file 9 **List of genes induced and repressed at 72 h in shGATA3 cells**. Genes were selected on a p-value of p < 0.01 and with an induction/repression cut-off values of 1.2 and 0.8, respectively. In the table, the expression ratio of irradiated versus non-irradiated cells is indicated. Click here for file
