# Supplementary figures and images for: GATA3 is a master regulator of the transcriptional response to low-dose ionizing radiation in human keratinocytes
Source: BMC Genomics. 2009 Sep 7;10:417. doi: 10.1186/1471-2164-10-417 (PMC2753551; doi:10.1186/1471-2164-10-417)

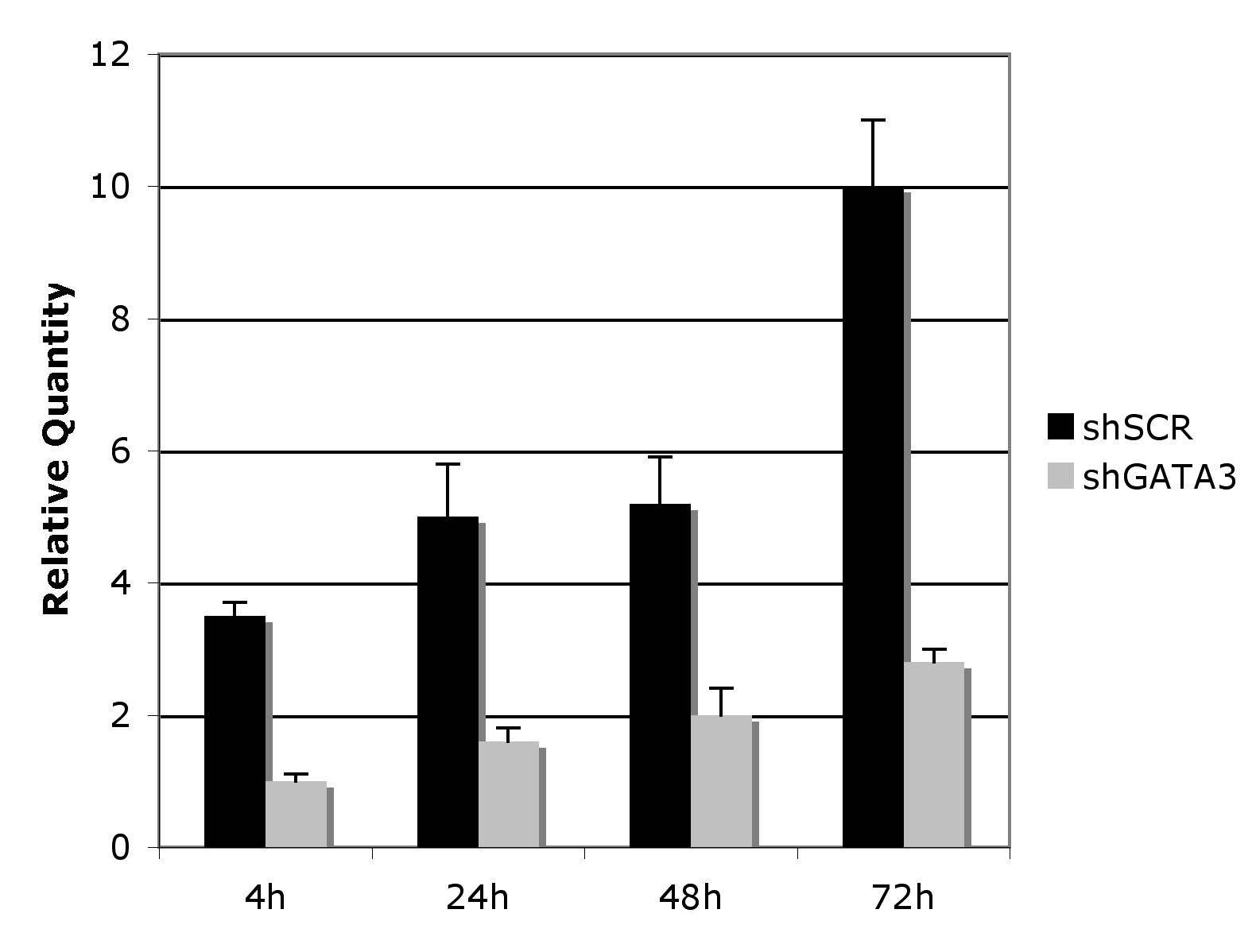

Supplement: Additional file 1 — Relative expression of GATA3 in shSCR and shGATA3 non-irradiated cells. Quantitative RT-PCR analysis of GATA3 transcripts at various time points after mock-irradiation of human keratinocytes with the various shRNA vectors. Errors bars correspond to standard deviations. [file 1471-2164-10-417-S1.png]
